# Effects of Time-Compressed Speech Training on Multiple Functional and Structural Neural Mechanisms Involving the Left Superior Temporal Gyrus

**DOI:** 10.1155/2018/6574178

**Published:** 2018-02-20

**Authors:** Tsukasa Maruyama, Hikaru Takeuchi, Yasuyuki Taki, Kosuke Motoki, Hyeonjeong Jeong, Yuka Kotozaki, Seishu Nakagawa, Rui Nouchi, Kunio Iizuka, Ryoichi Yokoyama, Yuki Yamamoto, Sugiko Hanawa, Tsuyoshi Araki, Kohei Sakaki, Yukako Sasaki, Daniele Magistro, Ryuta Kawashima

**Affiliations:** ^1^Faculty of Medicine, Tohoku University, Sendai, Japan; ^2^Division of Developmental Cognitive Neuroscience, Institute of Development, Aging and Cancer, Tohoku University, Sendai, Japan; ^3^Division of Medical Neuroimaging Analysis, Department of Community Medical Supports, Tohoku Medical Megabank Organization, Tohoku University, Sendai, Japan; ^4^Department of Nuclear Medicine and Radiology, Institute of Development, Aging and Cancer, Tohoku University, Sendai, Japan; ^5^Department of Functional Brain Imaging, Institute of Development, Aging and Cancer, Tohoku University, Sendai, Japan; ^6^Japan Society for the Promotion of Science, Tokyo, Japan; ^7^Division of Clinical Research, Medical-Industry Translational Research Center, Fukushima Medical University School of Medicine, Fukushima, Japan; ^8^Human and Social Response Research Division, International Research Institute of Disaster Science, Tohoku University, Sendai, Japan; ^9^Smart Ageing International Research Center, Institute of Development, Aging and Cancer, Tohoku University, Sendai, Japan; ^10^Department of Psychiatry, Tohoku University Graduate School of Medicine, Sendai, Japan; ^11^School of Medicine, Kobe University, Kobe, Japan

## Abstract

Time-compressed speech is an artificial form of rapidly presented speech. Training with time-compressed speech (TCSSL) in a second language leads to adaptation toward TCSSL. Here, we newly investigated the effects of 4 weeks of training with TCSSL on diverse cognitive functions and neural systems using the fractional amplitude of spontaneous low-frequency fluctuations (fALFF), resting-state functional connectivity (RSFC) with the left superior temporal gyrus (STG), fractional anisotropy (FA), and regional gray matter volume (rGMV) of young adults by magnetic resonance imaging. There were no significant differences in change of performance of measures of cognitive functions or second language skills after training with TCSSL compared with that of the active control group. However, compared with the active control group, training with TCSSL was associated with increased fALFF, RSFC, and FA and decreased rGMV involving areas in the left STG. These results lacked evidence of a far transfer effect of time-compressed speech training on a wide range of cognitive functions and second language skills in young adults. However, these results demonstrated effects of time-compressed speech training on gray and white matter structures as well as on resting-state intrinsic activity and connectivity involving the left STG, which plays a key role in listening comprehension.

## 1. Introduction

Listening comprehension is a complex cognitive act involving the maintenance of heard sentences with concurrent understanding. Even experienced nonnative listeners often have significant problems with listening comprehension of a second language compared with native listeners [1]. Furthermore, even experienced nonnative listeners benefit from the slowing down of auditory stimuli of the second language [2]. In this study, we focused on a training method known as time-compressed speech to tackle this problem.

Time-compressed speech is an artificial form of rapidly presented speech. As the speech rate is increased, comprehension accuracy generally declines [3]. Training with time-compressed speech of a second language (TCSSL) leads to adaptation toward TCSSL as well as different languages [1, 4].

During listening comprehension, the superior to middle temporal gyrus; the prefrontal areas, including the left inferior to superior frontal gyrus; and the premotor area are activated [5]. Regions in the bilateral superior to middle temporal gyrus and bilateral frontal operculum increase activity when subjects have to listen and comprehend auditorily presented fast sentences [5]. Adaptation to time-compressed speech appears to correspond with changes in brain activity of the superior temporal cortex and left premotor area during listening comprehension [5]. Among these bilateral superior temporal areas, it has been suggested that the left responds to linguistic content, whereas the right responds to complex auditory content [5].

On the other hand, the effects of cognitive training on untrained cognitive tasks as well as neural systems have been the focus of recent research in this field, which has included training with working memory [6–8], multitasking [9, 10], and processing speed [11]. These studies have demonstrated transfer effects of such training on cognitive functions (though, the effect size was shown to be modest through the meta-analyses [7, 8]) and effects of such training on neural systems and have suggested that one condition for the transfer effect to occur is that both training and transfer tasks engaged overlapping neural systems [12].

However, despite these studies, some issues remain unknown specifically (1) the extent of transfer of training with TCSSL, including diverse cognitive functions, and (2) whether training with TCSSL leads to the activation of neural mechanisms that are not task-specific. The purpose of this study was to investigate these issues. In particular, among a number of neural mechanisms, we focused on the microstructural property (fractional anisotropy) of white matter structure, which is known to play key roles in cognitive speed [13], and functional connectivity during rest (resting-state functional connectivity (RSFC)), which is affected by structural connectivity; fractional amplitude of spontaneous low-frequency fluctuations (fALFF), which has recently been associated with language-related abilities and skills in multiple relevant areas [14, 15], and which is shown to be sensitive to neural plasticity [16]; and regional gray matter volume (rGMV). Through these methods, a multimodal characterization of neural mechanisms underlying training with time-compressed speech of a second language becomes possible.

We hypothesized that training with TCSSL leads to the change of neural mechanisms involving the left superior temporal gyrus (STG) and possibly other frontal language-related areas, such as the left premotor area and left inferior frontal gyrus. As described above, the left STG is involved in the linguistic aspect of the comprehension of and adaptation to time-compressed speech and involvement of the left inferior frontal gyrus in the comprehension of time-compressed speech, whereas the left premotor area is involved in adaptation to time-compressed speech. More specifically, we expected the following neural changes: (a) changes to structural connectivity of the left arcuate fasciculus (AF) because this bundle is connected to the left STG and frontal language-related areas; (b) RSFC between the left STG and left frontal language-related areas; and (c) fALFF/rGMV in the left STG and left frontal language-related areas. Although this investigation of the effects on cognitive functions and skills was exploratory in nature, we expected to find benefits in (a) normal reading and listening skills of a second language because even experienced nonnative listeners benefit from slowing down of auditory stimuli of a second language [2], and therefore, we expected that adaptation to faster auditory stimuli would benefit normal listening skills of a nonnative listener; (b) reading comprehension of first and second languages, provided the overlap of the neural bases of reading and listening comprehensions [17] and the theory that one condition of the transfer effect is that both the training and transfer tasks engage overlapping neural systems [12]; (c) cognitive speed, because we expected that adaptation to faster auditory stimuli would generally benefit cognitive speed; and (d) working memory performance, provided that the abovementioned systems of listening comprehension overlap those of working memory [18] and the abovementioned theory of cognitive training transfer.

As described, considering the difficulty of second language listening comprehension, even for experienced nonnative listeners [2], and the diverse functions of the neural system for listening comprehension, it is important to investigate the extent of the plasticity achieved by training with TCSSL.

## 2. Methods

### 2.1. Subjects

The study cohort included healthy, right-handed undergraduate or graduate students with normal vision and no history of neurological or psychiatric illness, as assessed with a routine questionnaire in which each subject answered questions about a history of certain illnesses. Handedness was evaluated using the Edinburgh Handedness Inventory [19]. In this experiment, the inclusion criteria included English skills (EIKEN, an English proficiency assessment that is widely used in Japan), Grade Pre-2 (mid–high school level or greater), or a TOEIC score of >400. Written informed consent was obtained from each subject, and the study protocol was approved by the Ethics Committee of Tohoku University and performed in accordance with the tenets of the Declaration of Helsinki (1991).

Based on random allocation, 30 participants (12 men and 18 women) with a mean age of 20.7 ± 1.5 years were allocated to the training with TCSSL group. Thirty participants (16 men and 14 women) with a mean age of 20.8 ± 1.7 years were allocated to the active control group. None of the participants in the active control group were notified that they belonged to the “control” group until after the postmagnetic resonance imaging (MRI) and psychological examinations. For further complicated details of this experiment, refer to Supplemental Methods available here.

### 2.2. Procedure

Subjects underwent training for approximately 4 weeks (27 days) for 30–60 min each day in most cases. In both groups, the subjects used the auditory English stimuli files provided to them on their personal computers, and both groups were instructed to perform the task at home every day. The subjects were allowed to miss a session because of computer problems, illness, or other reasons and could complete the task more than once per day. The tasks for subjects in both groups included listening to each English auditory stimulus and recording their responses to the tasks in their computer files. The details of the task are described in the Supplemental Methods. Almost every day, the subjects uploaded the logs to the shared folders for compliance verification. The experimenter provided training feedback to the subjects as necessary (such as contacting the subjects when there was no upload for a while). MRI and psychological examinations were performed immediately before and after the 4-week training course. More specifically, pretraining MRI scans and psychological tests were performed on day 1, training was provided from day 2 to day 28, and posttraining MRI scans and psychological tests were performed on day 29.

### 2.3. Training Tasks

Some auditory English stimuli files that were appropriate for subjects' English skills were made available to the subjects on each training day. Each auditory English stimuli file took approximately 10 min for completion. The training with TCSSL group was given six files and the control group was given three files every day. Subjects were instructed to listen to the English stimuli files on their PC or tablets and perform the tasks for which instructions were given.

(a) In the training with TCSSL group, the playing speed was modulated according to the performance of the task. (b) In the control group, the pitch was modulated according to the performance of the task such that subjects had to listen to the English auditory files as high in pitch as possible while maintaining their task performance. For extensive details of the rationales of these procedures, see Supplemental Methods.

#### 2.3.1. Psychological Outcome Measures

For the evaluation of pre- and posttraining, a battery of neuropsychological tests and questionnaires was administered. For the rationales for the choice of tests and details of how tasks were administered, see Supplemental Methods. For details of each test, see each reference. This battery included the following contents: (A) Raven's Advanced Progressive Matrices [20], which is a nonverbal reasoning task. (B) A (computerized) digit span task, a verbal WM task (for the detail of this task, see [21]). (C) The Stroop task (Hakoda's version) [22, 23], which involves the word-color and color-word tasks as measures of simple processing speed and the Stroop and reverse-Stroop tasks as measures of inhibition. (D) Simple and complex arithmetic tasks [24] that measure simple and complex arithmetic abilities. (E) The SA creativity test [25], which measures creativity through divergent thinking [24]. (F) A Japanese reading comprehension task (for more details, see [26]). (G) A listening span task, for which simple sentences that state facts are presented successively, and the subjects are instructed to judge whether the sentences are correct and to remember the words that were at the start of each sentence. Each level has three trials, and the levels increase from 2 to 7. The number of trials in which the subjects remembered all the words in the correct order and judged the correctness of all sentences were recorded and used for analyses. (H) the TOEIC exam [27] to test English reading and listening. For the details, see Supplemental Methods.

#### 2.3.2. Image Acquisition

MRI data acquisition was performed using a 3T Philips Achieva scanner (Philips Healthcare, Andover, MA, USA). Diffusion-weighted data were acquired using a spin-echo planar imaging (EPI) sequence for FA analyses. For the details, see our previous study [28]. For the resting-state fMRI, 34 transaxial gradient-echo images covering the entire brain were acquired using an echo planar sequence. For this scan, 160 functional volumes were obtained while subjects were resting. For the details, see our previous study [29]. High-resolution T1-weighted structural images (240 × 240 matrix, TR = 6.5 ms, TE = 3 ms, FOV = 24 cm, slices = 162, and slice thickness = 1.0 mm) were collected using a magnetization-prepared rapid gradient echo sequence.

### 2.4. Preprocessing of Imaging Data

Preprocessing of regional gray matter structural data was performed using SPM12 implemented in Matlab. We obtained images of changes in the gray matter following training based on the longitudinal VBM method that uses the pairwise longitudinal registration toolbox [30] (smoothing: 8 mm FWHM) as was described previously [31]. For the VBM procedures, we used the same parameters as those used in our previous study [32].

Preprocessing of other imaging data was performed using SPM8 implemented in Matlab and the SPM8 extension software DPARSF (Data Processing Assistant for Resting-State fMRI). In the following procedures, coregistration and conormalization of pre- and postimages were avoided for the same reasons as described previously [10]. For rationales of this procedure, see Supplemental Methods. For RSFC analyses, in this study, we examined correlations associated with the area in the left STG, which is the main crux of the hypothesis, as described in the Introduction. The seed region of the left STG was a sphere with a 6 mm radius centered on the focus. The peak voxel of the left STG was *x*, *y*, *z* = −60, −14, −2; this voxel was shown to be active in response to difficult speech comprehension and showed the strongest effect in the meta-analysis [33].

#### 2.5. Statistical Thresholds for Group Level Analysis of Imaging and Behavioral Data

The behavioral data were analyzed using the Statistical Package for the Social Sciences (SPSS) 22.0 (IBM-SPSS Inc., Chicago, IL, USA). In our behavioral analyses, posttraining test scores in the training with TCSSL group were compared with those in the control group using one-way analyses of covariance (ANCOVAs), with pretest scores as covariates (*P* < 0.05). When there were hypotheses, one-tailed *t*-tests were used, as described previously [34, 35].

For group-level imaging analyses, during each whole brain analysis of RSFC, fALFF, and FA, posttraining values of each voxel in the training with TCSSL group were compared with those in the control group using one-way analyses of covariance (ANCOVAs), with the pretraining value of each voxel scored as a covariate. To conduct these analyses, we used biological parametrical mapping (BPM) [36] implemented in SPM8.

Analyses of the RSFC and fALFF were limited to the areas of the whole brain mask [29], and analyses of the FA were limited to the areas of the white matter mask of the DTI [37]. However, for FA analyses, in which we could not find significant findings in the whole brain analysis, small volume correction (SVC) was applied in the white matter tract of interest. Here, the white matter tract of interest was the left AF. To construct the mask images of the left AF, a DTI-derived atlas (http://www.natbrainlab.com/) was used [38]. Only clusters with *P* < 0.05 after correction for multiple comparisons at a cluster size with an uncorrected voxel-level cluster-determining threshold of *P* < 0.001 were considered statistically significant in these analyses.

In the group-level imaging analysis of rGMV, using the one-way ANOVA option in SPM8, we tested for group-wise differences in the change in rGMV at each voxel across the whole brain. This analysis was limited to areas where the average rGMV value for the segmented and normalized mean images of pre- and postscans of all participants was greater than 0.1. A multiple comparison correction was performed using threshold-free cluster enhancement (TFCE) [39] with randomized (5000 permutations) nonparametric testing using the TFCE toolbox (http://dbm.neuro.uni-jena.de/tfce/). We applied a threshold of family-wise error corrected at *P* < 0.05.

For rationales for using different statistical tests for rGMV, see Supplemental Methods.

## 3. Results

### 3.1. Differences in the Characteristics of the Two Training Groups

Before the intervention period, there were no significant differences in the demographics among the groups (*P* > 0.05, two-tailed *t*-tests), such as age (*P* = 0.812, *t* = −0.238); sex (*P* = 0.309, *t* = 1.027); score of the Raven's Advanced Progressive Matrix [20], which measures cognitive ability, which is central to general intelligence [40] (*P* = 0.082, *t* = −1.770); or scores of the English listening (*P* = 0.331, *t* = −0.980) and reading tests (*P* = 0.832, *t* = 0.213) (described below).

One participant in the training with TCSSL group and another in the active control group withdrew from the study after the pre-MRI and psychological experiments and did not participate in the post-MRI and psychological experiments. Thus, 29 subjects in the training with TCSSL group and 29 subjects in the active control group successfully completed the study.

Furthermore, according to the questionnaire that was administered to participants after the experiments, there was no significant difference between the two groups in motivation toward the training tasks (*P* = 0.539, *t* = 0.618), fatigue of the subjects after the training tasks (*P* = 0.461, *t* = −0.743), fatigue of the subjects after the training period (*P* = 0.395, *t* = −0.858), satisfaction with the training tasks (*P* = 0.930, *t* = 0.088), enjoyment during the training tasks (*P* = 0.948, *t* = −0.065), and expectations of effects of training on English listening (*P* = 0.634, *t* = 0.479) and reading (*P* = 0.210, *t* = 1.270) skills (*P* > 0.2, uncorrected).

### 3.2. Training Data

During the 27-day intervention period, the mean number of sessions completed by the subjects in the training with TCSSL group was 25.48 ± 2.79 and 25.97 ± 2.39 for subjects in the active control training group. Given that one subject could not complete more than 27 sessions, these results show that the subjects were mostly able to perform the task properly. The level of performance (the highest difficulty level at which the subjects achieved at least five correct answers to the 10 problems in each set of auditory stimuli) was significantly increased in the last three training sessions compared with the first three training sessions (paired *t*-test, *P* < 0.001, Table 1) in both groups. The fact that subjects in the active control group (which changed the pitch of the stimuli) faced this limitation suggests that when the pitch of the stimuli is increased, the subjects become unable to comprehend the stimuli.

### 3.3. The Effect of Training with TCSSL on Psychological Test Performance

Analyses of the psychological tests revealed no significant differences in pre- to posttest changes in performance between the active control group and the training with TCSSL group (for all the results of the psychological measures, see Table 2). This result cannot be explained by preexisting differences (if any) between groups because the pretest scores were controlled by ANCOVAs in these analyses.

In these analyses, subjects who misunderstood the rules of the tasks (four subjects in the listening span tasks and one subject in the Stroop tasks) or whose data was missing (two subjects in the English reading task and one subject in the English listening task) were excluded from the analyses using those measures.

### 3.4. The Effect of Training with TCSSL on fALFF

**Figure 1:**
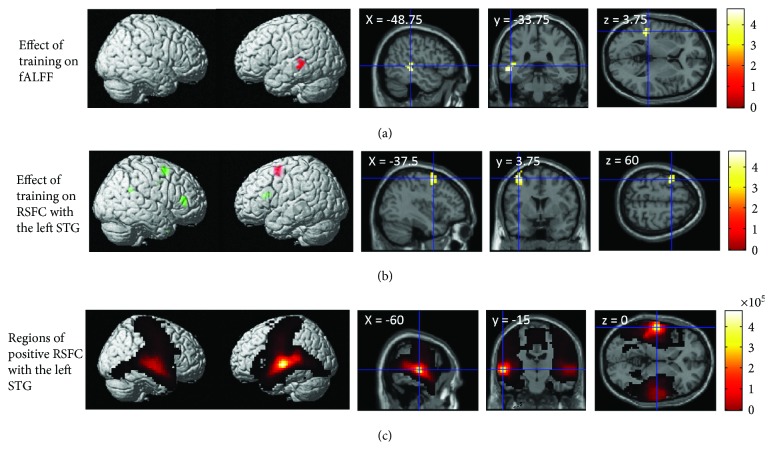
The effect of training with TCSSL on RS-fMRI measures. (a) The effect of training with TCSSL on fALFF. The results are shown with *P* < 0.05, corrected for multiple comparisons at cluster-level with an underlying voxel-level of *P* < 0.001, uncorrected. There was a larger increase in fALFF in the training with TCSSL group compared with the active control group (this analysis was performed to identify differences in pre- to posttraining changes between groups, as described in the Methods section). Compared with active control training, training with TCSSL resulted in an increase in fALFF in the left STG and the left middle temporal gyrus. (b) The effect of training with TCSSL on RSFC with the left STG. There was an increase in RSFC with the left STG in the training with TCSSL group compared with the active control group (red areas: *P* < 0.05, corrected for multiple comparisons at cluster-level with an underlying voxel-level of *P* < 0.001, uncorrected; green areas: *P* < 0.001, uncorrected). Compared with the active control training, the training with TCSSL resulted in a significant increase (red areas) in RSFC between the left STG and an anatomical cluster that spread around the left middle frontal and superior frontal premotor area (this analysis was performed to identify differences in pre- to posttraining changes between groups, as described in the Methods section) and tendencies of increase in RSFC with the left STG in bilateral frontal and right temporal areas. (c) Regions that showed positive RSFC with the left STG. The results shown were obtained using a threshold of threshold-free cluster enhancement (TFCE), *P* < 0.05 based on 5000 permutations.

After correcting for confounding variables, whole brain analysis revealed that, compared with the active control group, the training with TCSSL group showed a significantly greater pre- to post-MRI experiment change of fALFF in the anatomical cluster that mainly spread in the posterior part of the left STG and left middle temporal gyrus (Figure 1; *x*, *y*, *z* = −52.5, −30, −3.75; *t* = 4.74; *P* = 0.009, corrected for multiple comparisons at the cluster level with a cluster-determining threshold of *P* < 0.001, uncorrected, 1477 mm^3^). The mean fALFF value of this cluster significantly increased from pre- to postexperiment in the training with TCSSL group (*P* < 0.01, paired *t*-test). The identified area is distant from the peak voxel of the seed area of the RSFC analysis (*x*, *y*, *z* = −60, −14, −2). However, when we looked at the result of group comparisons of fALFF change with a more lenient threshold (*P* < 0.05, uncorrected), an extensive cluster was observed in the left STG overlapping with the seed area of the RSFC. Thus, segregation of the areas was unclear. There were no significant areas where the training with TCSSL group showed a significantly greater pre- to post-MRI experiment decrease of fALFF compared with the active control group.

### 3.5. The Effect of Training with TCSSL on RSFC with the Left STG

Next, we compared changes in RSFC with the area of the left STG, which plays a key role in difficult speech comprehension (the seed region), in the training with TCSSL and control groups. We found that the training with TCSSL group showed a significantly greater pre- to post-MRI experiment increase of RSFC between the left STG (the seed region) and an anatomical cluster that spread around the left middle frontal and superior frontal premotor areas (Figure 1; *x*, *y*, *z* = −37.5, 3.75, 60; *t* = 4.93; *P* = 0.029, corrected for multiple comparisons at the cluster level with a cluster-determining threshold of *P* < 0.001, uncorrected, 2162 mm^3^). The mean *Z* value for this cluster was not significantly different from 0 in the training with TCSSL group preexperiment (*P* > 0.2), but was significantly greater than 0 postexperiment (*P* < 0.01). The mean *Z* value of this cluster significantly increased from pre- to postexperiment in the training with TCSSL group (*P* < 0.01, paired *t*-test). This means that the relative change in RSFC in the training with TCSSL group may be interpreted as an expansion of the areas that have positive RSFC with the seed region. There were no significant areas where the training with TCSSL group showed a significantly greater pre- to post-MRI experiment decrease of RSFC with the area of the left STG compared with the active control group.

The fALFF and rGMV analyses described later both showed the more posterior part of the left temporal cortex. Thus, post hoc analyses showed that the RSFC with the seed region of interest (ROI) of the peak voxel of the posterior part of the temporal gyrus (*x*, *y*, *z* = −58, 46, 4) was active in response to difficult speech comprehension (taken from the meta-analysis of [33]) in a study with the same statistical design. This analysis revealed a similar tendency of a relatively larger increase in RSFC in the training with TCSSL group in a similar premotor area (*x*, *y*, *z* = −37.5, 11.25, 52.5; *t* = 3.44, uncorrected, *P* < 0.001). In this analysis, no other significant results were observed, although, in the area close to the right anterior cingulate cortex, there was a significantly larger RSFC increase in the training with TCSSL group in comparison with the control group, but only when the voxel level multiple comparison correction was applied (*x*, *y*, *z* = 18.75, 11.25, 30; *t* = 5.27, *P* = 0.045, corrected for multiple comparisons using voxel-level family-wise error).

### 3.6. The Effect of Training with TCSSL on FA

**Figure 2:**
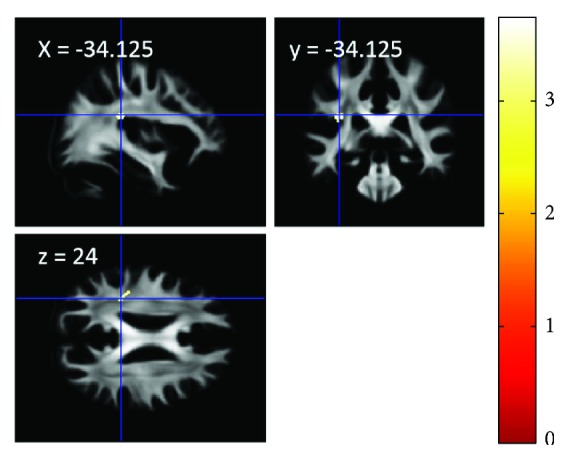
The effect of training with TCSSL on FA. The results are shown with *P* < 0.05, corrected for multiple comparisons at cluster-level within the area of the left AF with an underlying voxel-level of *P* < 0.001, uncorrected. There was a larger increase in FA in the training with TCSSL group compared with the active control group. Compared with the active control training, the training with TCSSL resulted in an increase in FA of an area in the left AF (this analysis was performed to identify differences in pre- to posttraining changes between groups, as described in the Methods section). Regions showing a significant effect were overlaid on mean preprocessed, but not smoothed, FA images of the participants.

After correcting for the preintervention FA value at each voxel, whole brain analysis revealed that there were no significant differences in pre- to post-MRI experiment FA changes between groups. However, SVC analysis of the left AF revealed that training with TCSSL group showed a significantly greater pre- to post-MRI experiment increase in FA in the anatomical cluster in the left AF (Figure 2; *x*, *y*, *z* = −33.25, −35, 22; *t* = 3.71; *P* = 0.010, corrected for multiple comparisons within the left AF at the cluster level with a cluster-determining threshold of *P* < 0.001, uncorrected, 214 mm^3^). The mean FA value of this cluster significantly increased from pre- to postexperiment in the training with TCSSL group (*P* < 0.01, paired *t*-test).

There were no significant areas where the training with TCSSL group showed a significantly greater pre- to post-MRI experiment decrease of FA compared with the active control group.

### 3.7. The Effect of Training with TCSSL on rGMV

**Figure 3:**
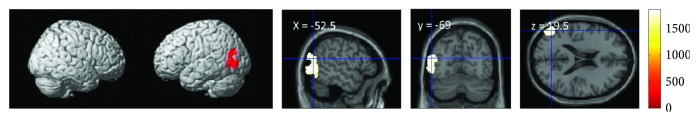
The effect of training with TCSSL on rGMV. The results shown were obtained using a threshold of threshold-free cluster enhancement (TFCE), *P* < 0.05 based on 5000 permutations. There was a greater decrease in rGMV in the training with TCSSL group compared with the active control group (this analysis was performed to identify differences in pre- to posttraining changes between groups, as described in the Methods section). Compared with the active control training, the training with TCSSL resulted in a decrease in rGMV in the junction of the left middle and STG and the left middle and superior occipital gyri.

Voxel-based morphometry analysis revealed that, compared with the active control group, the training with TCSSL group showed a statistically significant larger training-related decrease in the rGMV of the junction of the left middle and STG and left middle and superior occipital gyri (Figure 3; *x*, *y*, *z* = −55.5, −78, −1.5; TFCE value = 1865.79; *P* = 0.032, corrected for multiple comparisons using TFCE (family wise error), 8619.75 mm with the threshold of *P* < 0.05, corrected for multiple comparisons using TFCE). The mean rGMV change value from pre- to postexperiment of this cluster was significantly greater than 0 in the training with TCSSL group (*P* < 0.01, paired *t*-test).

The identified area where there was a difference of rGMV change between the groups did not overlap with that of fALFF described above. However, when we looked at the results of group comparisons of fALFF change and those of rGMV change with a more lenient threshold (*P* < 0.05, uncorrected), the two effects substantially overlapped. Thus, the segregation of the two effects was unclear. However, with this lenient threshold, the cluster where there was an effect of group difference of rGMV still did not overlap with the seed area of the RSFC (the more anterior part of the left STG).

There were no significant areas where the training with TCSSL group showed a significantly greater pre- to post-MRI experiment increase in rGMV compared with the active control group.

## 4. Discussion

The present study investigated the effect of training with TCSSL on diverse cognitive functions and neural systems. The results showed that, compared with active control training, training with TCSSL led to an increase in fALFF of the left STG, an increase in RSFC between the area that plays a key role in difficult speech comprehension and the premotor area, an increase in FA in the left AF, and a decrease in rGMV of the temporal and occipital junction area involving the left STG. These results were at least partially mediated by the pre- to postchanges in the TCSSL group as shown by the results, which were partly consistent with our hypotheses set out in the Introduction, except that the obtained results of rGMV and fALFF did not involve frontal language-related areas. On the other hand, contrary to our hypotheses, there were no significant differences in the changes in the performance of measures of cognitive function or second language skills after training with TCSSL compared with active control training.

The results of this study have advanced our understanding of adaptation to time-compressed speech. As described in the Introduction, it was previously shown that training with TCSSL leads to adaptation toward TCSSL (meaning one becomes able to comprehend more fluently) and time-compressed speech in different languages [1, 4] as well as to changes in neural activity in response to time-compressed speech as the subject adapts to time-compressed speech [5]. The present study investigated the effects of training with TCSSL on second language skills and diverse cognitive functions as well as task-free or stimuli-free neural mechanisms. We revealed that training with TCSSL went beyond neural changes in response to stimuli of time-compressed speech and led to diverse structural and resting-state imaging measures.

The fALFF increase in the left STG and MTG may reflect increased default activity of the areas involved in semantic and language comprehension. This area is involved in sentence comprehension and difficult sentence comprehension, and neural activity in response to time-compressed speech was shown to decrease with adaptation to time-compressed speech [5]. It has been suggested that the STG plays key roles in semantic aspects of comprehension [41], regardless if the language is the listener's first or second language [42]. When the speed of the auditorily presented sentence is increased, activation of this area increases, but when the speed is too fast and the sentence is no longer comprehensible, the activation of this area decreases to the baseline level [43]. But, as the subjects become accustomed to the fast auditory presentation of the sentence and the subjective level of comprehension difficulty decreases, activation of this area decreases to the levels observed for sentences presented at a normal speed [5]. There was a significant positive correlation between fALFF in the left MTG and semantic processing ability [14]. fALFF is the amplitude of BOLD signal fluctuation and is assumed to indicate the magnitude of neural activity during rest [44–46]. Further, the metabolic demands of the brain are largely accounted for by the amplitude of brain activity [47]. In addition, a previous study reported that comprehensive intervention involving cognitive training, exercise, and counseling resulted in an increase in the ALFF of the prefrontal areas and cerebellum and led to changes in cognitive function and well-being, and changes of ALFF were associated with these cognitive changes [16]. Moreover, it was suggested that resting-state ALFF is a marker of intervention-induced plasticity [16]. Thus, the present study showed that midterm training with TCSSL resulted in increased default activity in regions involved in semantic and text comprehension.

Increased RSFC between the area of the left STG and the premotor areas involving the superior frontal and middle frontal gyri may reflect increased efficiency of information transfer regarding language comprehension. The seed ROI set in this study was the area involved in difficult speech comprehension [33]. On the other hand, the identified premotor areas are spatial areas of working memory that correspond to areas involved in semantic and sentence comprehension as well, although they may also be adjacent to areas involved in phonological processing (for review, see [48]). Furthermore, it has been shown that the RSFC between the middle temporal area and the left premotor area is associated with semantic processing ability [14]. The seed ROI and the left premotor area both overlap with areas involved in time-compressed speech comprehension, and activation of these areas changes with adaptation to time-compressed speech [5]. Thus, the observed increase in RSFC between two regions may reflect the increased connectivity between the frontal and temporal areas involved in sentence comprehension. However, it has been suggested that adaptation to TCSSL involves premotor neural changes associated with phonological processing [49], and these observed changes may also involve the neural changes involved in phonological processing.

With respect to the study by Wei et al. [14], although a number of neural changes involving the left temporal areas were observed in response to training with TCSSL, no transfer effects were observed in the performance of cognitive tests because there were no improvements in English listening and reading skills. Although the reason for this divergence is unclear, we speculate that regarding English listening and reading skills, each of the subjects had a long history of English instruction and a considerable knowledge of grammar; thus, an extended vocabulary was required to achieve good scores on these general tests. The listening comprehension tests used here may not particularly accentuate the speed in setting the difficulty, and just 20 h of specific additional training may have been insufficient to improve such performance. Similarly, for one to gain higher cognitive function, neural changes in a wide range of areas may be required [50]. On the other hand, our battery of cognitive tests did not include measures of semantic processing ability, such as those used by Wei et al. [14]. Thus, future studies should investigate whether training of TCSSL or other training that causes change to multiple neural mechanisms in the STG and MTG can result in transfer to semantic processing ability.

The FA increase in the training with TCSSL group in the left AF may reflect myelination processes that made it possible to adapt to faster speech comprehension. The left AF is involved in the transfer of speech/language information from posterior brain areas to premotor/motor areas, and it has been suggested that it this process plays important roles in language processing [51]. Increased FA changes through neural plasticity have been assumed to reflect an increase in myelination processes which continue throughout adulthood [52]. The myelination process was affected by and could be manipulated from the outside within a week [53] as well as could be affected by environmental influences [54]. It is well established that the myelination process continues even into adulthood [55–57]. An action potential spreads faster along myelinated axons than along unmyelinated axons [58] and along axons with thicker myelin [59]. A faster conduction velocity can facilitate information flow not only by speeding it up but also by allowing for precise temporal coding of high-frequency bursts of neuronal activity [60]. Thus, through these processes in the white matter bundle, which is critical to language processing, adaptation to faster speech may occur. Furthermore, these structural changes may underlie the RSFC changes described above [61]. But, as with most macro-level neuroimaging measures, FA measures are affected by multiple physiological processes [62]. Thus, our speculation in this paragraph must be confirmed in future studies.

The present results suggest that training with TCSSL led to a decrease in rGMV, although the reason for this change remains unclear. In our previous study [24], we speculated that the usage-dependent selective elimination of synapses [63] underlies the decreases in rGMV. Selective elimination of synapses is known to sculpt neural circuitry [64]. In support of this theory, a decrease in rGMV after cognitive training or other interventions is sometimes observed [24, 34, 65, 66]. However, mild mid-term training mainly leads to an increase in rGMV [10, 67–70]. Previously, based on observations from a number of studies, we proposed that these differences may be explained by nonlinear changes in rGMV (an initial increase followed by a decrease), which are affected by the length and intensity of training [24]. However, in previous studies, training with an intensity and duration similar to those of the present study led to an increase in rGMV [10, 70]. It has been shown that learning new processes can lead to a transient increase in spine formation and that this rapid spinogenesis is followed by enhanced spine elimination [71]. However, the elimination during subsequent training is largely restricted to spines that existed before training [71]. Perhaps, listening by those well-experienced in the second language largely employed the spines that existed before training and may lead to a phase where elimination becomes dominant faster. But, these theories are purely speculative and should be verified in future animal studies. Currently, the factors that lead to an increase or decrease in rGMV remain unclear.

Changes in rGMV and fALFF were observed in the more posterior part of the left STG, suggesting neural changes related to the semantic process. An extensive area in the left STG is active during speech comprehension. However, the anterior part of the left STG may be more involved in the auditory comprehension of internal and external verbal stimuli (e.g., internal speech and auditory speech) as well as internal and external acoustic stimuli, whereas the more posterior area of the left STG may be more involved in the semantic process [72]. Among areas of the left STG, neural changes related to these semantic processes may occur more strongly, at least with regard to rGMV and the amplitude of intrinsic brain activity.

This study had a few limitations that should be addressed. First, we did not establish a passive control group and we compared the effects of training with TCSSL with the active control training. Although the active control training was designed to be ineffective, it is not known whether this was truly the case. Nonetheless, the effects of training with TCSSL were compared with those of active control training. Further, we could not control the amount of English that subjects in each group received in this experiment. This type of intervention study requires many resources, and it is difficult to create control groups for all confounding factors. In this experimental design, when time for training is relatively controlled, the amount of exposed English stimuli cannot be controlled. However, we believe that like other factors that we could not control (such as the pitch of the stimuli), the amount of English stimuli was not important in this study because only subjects with relatively high level of English skills and who studied the language for at least 6 years participated in this study. EIKEN (an English proficiency assessment that is widely used in Japan), Grade Pre-2 (middle level of high school) or higher, or a TOEIC score of >400 was a condition for participation in this experiment. Therefore, it would be surprising if receiving normal English auditory stimuli that are easy enough to comprehend at a normal speed for a short period of time would substantially affect neural mechanisms.

In summary, the present study investigated the effects of training with a second language on diverse cognitive functions and second language skills as well as neural mechanisms in young adults. Consistent with most of our hypotheses, our results confirm that training with TCSSL causes plasticity in multiple neural mechanisms involving the left STG. However, despite improvements in the performance of training tasks and adaptation to TCSSL, no transfer of training effects to behavioral measures of a wide range of cognitive functions were observed. The observed neural changes caused by training with TCSSL may offer new insights into how multiple neural mechanisms promote adaptation to time-compressed speech.

## Figures and Tables

**Table 1:** The mean ± SD difficulty level of the most difficult performances of all subjects (the highest difficulty level at which the subjects still achieved at least five correct answers to the 10 problems in each set of auditory stimuli in training tasks among the first three and last three training sessions).

	First three sessions	Last three sessions
Training with TCSSL (times faster than the original speed)	2.09 ± 0.38	2.97 ± 0.55
Active control training (semitones (#) higher than the original pitch)	12.72 ± 2.49	19.36 ± 5.32

**Table 2:** Pre- and posttest scores for psychological measures (mean ± standard error of mean).

	Training with TCSSL (experimental group)^b^		Active control		Planned contrast	*P* value^c^	Effect size (*d*)^d^
	Pretest scores	Posttest scores	Pretest scores	Posttest scores			
English listening test (score)	268.1 ± 53.46	252.24 ± 53.2	283.93 ± 47.25	269.64 ± 46.54	Experimental > control	0.884	−8.33 × 10^−3^
English reading test (score)	232.93 ± 57.98	221.72 ± 55.04	230.00 ± 60.29	238.7 ± 54.65	Experimental > control	0.938	−0.0282
RAPM^a^ (score)	26.61 ± 4.43	29.24 ± 4.16	28.79 ± 4.80	31.03 ± 4.67	Experimental > control	0.576	−2.09 × 10^−4^
Digit span (score)	37.17 ± 7.16	37.72 ± 8.69	38.90 ± 7.49	38.76 ± 7.10	Experimental > control	0.417	3.85 × 10^−4^
Listening span test (score)	12.79 ± 2.67	13.38 ± 3.03	12.68 ± 3.34	12.96 ± 2.86	Experimental > control	0.311	3.86 × 10^−3^
Word-color task (items)	67.69 ± 9.04	75.90 ± 8.22	72.28 ± 6.21	77.17 ± 6.53	Experimental > control	0.236	6.36 × 10^−3^
Color-word task (items)	52.97 ± 5.26	53.83 ± 7.49	53.48 ± 5.65	55.52 ± 6.94	Experimental > control	0.840	−6.05 × 10^−3^
Reverse Stroop task (items)	60.90 ± 6.06	63.69 ± 7.42	62.76 ± 5.58	65.34 ± 8.13	Two tailed	0.893	1.47 × 10^−4^
Stroop task (items)	48.72 ± 6.75	51.38 ± 7.14	51.76 ± 6.52	53.69 ± 7.14	Two tailed	0.688	8.53 × 10^−4^
Simple arithmetic (items)	31.93 ± 5.15	32.45 ± 6.17	32.52 ± 5.14	32.43 ± 4.54	Experimental > control	0.260	2.38 × 10^−3^
Complex arithmetic (items)	7.55 ± 4.45	7.90 ± 4.48	6.72 ± 2.18	7.53 ± 2.14	Experimental > control	0.859	−3.22 × 10^−3^
Japanese reading comprehension (items)	14.31 ± 4.42	18.38 ± 6.42	13.66 ± 3.76	18 ± 5.36	Experimental > control	0.633	−8.63 × 10^−4^
SA creativity test (total grade)	25.41 ± 6.12	24.31 ± 5.25	25.34 ± 5.55	25.34 ± 6.00	Two tailed	0.379	−9.01 × 10^−3^

^a^Raven's advanced progressive matrices; ^b^time-compressed speech training; ^c^one-way analysis of covariance with test-retest differences in psychological measures as dependent variables and pretest scores of the psychological measures as covariates; ^d^effect size estimates were calculated using Cohen's *d*.

## Supplementary Materials

Supplementary Materials Supplemental online material details of the design of the present experiment.
