# Supplementary material for: Effects of Time-Compressed Speech Training on Multiple Functional and Structural Neural Mechanisms Involving the Left Superior Temporal Gyrus
Source: Neural Plast. 2018 Feb 20;2018:6574178. doi: 10.1155/2018/6574178 (PMC5838482; doi:10.1155/2018/6574178)
Supplement: Supplementary Materials — Supplemental online material details of the design of the present experiment. [file 6574178.f1.docx]

**Supplemental Online Material**

**Details of the design of the present experiment**

The study cohort included 119 healthy, right-handed undergraduate or graduate students (67 men and 52 women; mean age, 20.6 ± 1.58 years)

This study was conducted together with another interventional study to investigate the effects of shadowing training and reading aloud training. Subjects in the active control group participated in both studies; All the groups went through the common, psychological and neuroimaging outcome measures, training period, and training frequency. Thus, the data of the active control group can also be used as a reference for analyses of shadowing training and reading aloud training. Participants were randomly assigned to one of the four groups: the training with TCSSL group, a shadowing training group, a reading aloud training group, or an active control group. Four interventions were performed in parallel, and this study design does not involve a crossover design and participants of each group were exposed to only one type of training and with no interventions subsequently.

Note that the subjects were students who were exposed to English in classes or other places, at least to some extent. However, this was a randomized controlled study and like any other numerous factors that can affect outcome measures, such points are not supposed to affect group differences of those outcome measures.

**Details and rationales of training tasks**

Some auditory English stimuli files from Voice of America Special English (http://learningenglish.voanews.com/), Nihon Housou Kyokai (NHK; Japan Brodacasting Cooperation) news (Torikai and Ito, 2013), and NPR (<http://www.npr.org/>) were made available to the subjects on each training day (the details are described below). The auditory English stimuli files from Voice of America Special English were used by the subjects with a beginner level of English, those from Nihon Housou Kyokai news by the subjects with an intermediate level of English, and those from National Public Radio news by the subjects with a high level of English. Levels of English comprehension were determined through preliminary investigations and classified according to the Test of English for International Communication (TOEIC) listening test (described later), with a score of 0–300 indicating a beginner level, 300–400 as an intermediate level, and 400–495 as a high level. A score was assigned simply to supply an appropriate level of difficulty at a normal speed. If the text was too difficult, then subjects were not able to comprehend the stimuli at a normal speed. If the text was too easy, then the task was not sufficiently challenging even when speeded up (within the limitation of the software and experimental procedure). It was difficult for us to gather the subjects who could participate in this intervention study, and it was not possible to further limit the subjects’ levels of comprehension within the small range. On day 1, the subjects undertook the TOEIC practice exam [1] and received auditory English stimuli files for training based on the score of this test. Each auditory English stimuli file took approximately 10 min to complete. The training with TCSSL group was given six files and the control group was given four every day. Subjects were instructed to listen to the English stimuli files on their PC or tablets and perform the instructed tasks.

(a) In the training with TCSSL group, the playing speed was modulated according to the performance of the task, as described below. We chose 10 English sentences (such as “Smartphones are ruining the concert-going experience”) from each English auditory file and translated each into Japanese. The subjects were given the text files comprising 10 of these translated Japanese sentences and assigned question numbers 1–10, for which the subjects were instructed to identify the English sentences that corresponded to the given 10 Japanese sentences while they listened to the auditory file and then asked to record their voice saying “Yes” when they found the given Japanese sentence. If the subjects were unable to identify the given sentence, they were asked to ignore the sentence and proceed to the next given sentence. In the English auditory file, the English sentences that corresponded to the given Japanese sentences appeared in numerical order (in other words, sentence “1,” sentence “2,” …, and sentence “10” appeared in this order in each English file). For the subjects to judge when the given 10 Japanese sentences appeared, 8 s after the English sentences that corresponded to the given Japanese sentences streamed, the sentence number streamed (“1,” “2,” and “3” and so on). In other words, 8 s after sentence “1” streamed, the spoken word “one” (in Japanese) streamed. If the subjects could record their voice saying “Yes” before the corresponding question number streamed, this established that they could identify the given Japanese sentence that corresponded to the question number and had found it correct. If the subject stated the word “Yes” at an inappropriate time, the response was regarded as incorrect and that the subject was unable to identify the correct answer. There were 10 sentences per English file, and each day the subjects in the training with TCSSL group listened to six English files and were instructed to complete the associated tasks.

The stimulus speed was modulated according to how well the subjects could detect the given text sentences using the free sound software Windows Media Player (Microsoft Corporation, Redmond, WA, USA). For the first trial on the first training day, the subjects were instructed to listen to the auditory English stimuli files at 1.5 times faster than the original speed. Subsequently, the stimulus speed was modulated based on the performance in the previous trial. If the subject’s performance (the number of the sentences that the subjects identified correctly) in the previous trial is expressed as X (which has a value between 0 and 10 as there were only 10 sentences in each trial) and the listening speed for that trial was Y_t_ (i.e., in the trial, the speed of the stimuli was Y_t_ times faster than the original speed), then the speed of the task in the subsequent trial (Y_t+1_) became [Y + 0.05 × (X − 5)], faster than the original speed. When the subjects’ performance was graded as a little bit good, the difficulty of the task was increased a little bit. Through this procedure, the speed was modulated finely. But, when the task was too easy or too difficult, the difficulty of the task was rapidly adjusted to an appropriate level. This procedure was used in our previous studies [2] and was found to effectively adjust the difficulty of the task. When the speed of the task becomes twice faster than the original speed, subjects were instructed to modulate the speed of the stimuli which were twice faster than the original speed. This kind of adaptive modulation of difficulty of task has been used in a wide range of training paradigms and shown to be useful and became the standard procedure in the field [3, 4].

(b) In the control group, the pitch was modulated according to the performance of the task such that subjects had to listen to the English auditory files as high in pitch as possible while maintaining their task performance. This training method was chosen because, changing the pitch is not known to cause the kind of plasticity that other training methods cause as described in Introduction. Also, neural systems respond to stimuli of different in a tonotopic manner [5], while the they respond to stimuli of faster speed by increasing activity [6].

The English auditory files were streamed, and the pitch was modulated using the free sound software Hayaemon (<http://soft.edolfzoku.com/hayaemon2/>). At first, the subjects were instructed to listen to auditory English stimuli files at 10 semitones (10#) higher than the original sound. One unit for modulation of the task difficulty was 0.5 semitone (#), and the pitch of the task was decreased or increased in a similar manner to the modulation of speed used for the training with TCSSL group, with the stimuli pitch modulated based on how well the subjects could detect the given text sentences. Specifically, if the subjects’ performance in the previous trial was X (a value between 0 and 10, as there were only 10 sentences in each trial) and the current listening pitch was Y (e.g., if Y = 12, subjects had to listen to auditory English stimuli files at 12 semitones (10#) higher than the original sound), then the pitch in the subsequent trial became [Y + 0.5 × (X − 5)].

The conditions for the stimuli files and required tasks were the same as those for the training with TCSSL group. However, the auditory files in the training with TCSSL group were run at a higher speed, resulting in a shorter duration. The active control group therefore listened to three English files each training day. Through these training methods, the active control group experienced similar stimuli, tasks, feedback on performance, and adaptive modulation of difficulty to the training with TCSSL group.

**Rationales for the choice of psychological tests and details of administering the tests**

The specific battery of cognitive tests in this study was chosen for this study for specific and unspecific theoretical and practical reasons. The theoretical and study-specific reasons included the hypotheses described in this study. The practical reasons included (a) availability in Japan, (b) ease to administer to several participants at once, and (c) use of common tests across the different intervention studies [e.g., 7] as much as possible to allow possible comparisons across studies (for reference) as well as for experimenters to recognize the pitfalls and characteristics of the cognitive tests before the study.

Several questionnaires designed to assess the traits or states of the subjects were collected but are not described in this study. Other than the self-reported questionnaires, all neuropsychological assessments were performed by postgraduate and undergraduate students who were blinded to the group membership of the participants.

**Details of the TOEIC exam.**

The TOEIC exam [1] to test English reading and listening. We used the listening and reading tests of this practice exam. The listening test consists of four parts that the subjects can hear only once, and the subjects respond to a total of 100 questions. The entire listening test lasts for approximately 45 min. The subjects hear the dialogue of the question and are then instructed to choose the correct answer about the dialogue from several options. The reading test, which takes approximately 60 min to complete, consists of three parts, for which the subjects read a short or long text and then choose an appropriate answer for a total of 100 questions.

**The reason for independent normalizations of pre- and post-intervention brain images**

The reason for independent normalization is described in our previous study [8] and reproduced as follows. We avoided co-registration and co-normalization procedures (which use the same normalization parameters for both the pre- and post-images) of pre- and post-images, including registration of mean images to pre- and post-images, because of concerns of possible bias or problems occurring when structural properties were substantially different between pre- and post-images [9].

**The rationales of some of procedures of RSFC analyses.**

For RSFC analyses, 27 nuisance covariates, including mean signals from the voxels within the white matter mask, mean signals from the voxels within the CSF mask, mean signals from the voxels within the whole brain mask, and Friston 24 motion parameters, were regressed out. In this study, we regressed the whole brain signal out in the RSFC analyses because, as we discussed earlier, the mean whole brain signal partly reflects global brain activity [10] and we wished to observe network-specific activities in the case of RSFC. In the fALFF analyses, which are described below, this was not necessarily the case (meaning, we would like to reveal the global brain activity change, too if there is one); therefore, global brain activity was not regressed out. For more details and rationales for these procedures, see our previous study [11]. Nonetheless, a similar tendency still remained by regressing out the global brain activity of the significant results of the fALFF analyses in the left temporal gyrus reported in the Results section (in the case when the global signal is regressed out, the t-value became 4.01 and the cluster size was 52.7 mm^3^, with the threshold of uncorrected *P* < 0.001).

After preprocessing, the fMRI data were temporally band-pass filtered (0.01 < f < 0.08 Hz) to reduce low and high frequency drift. This bandpass filter (0.01–0.08Hz) is widely used in the field [12] because it can cut frequencies of possible confounding cardiac (∼0.8–1.0 Hz) and respiratory (∼0.3 Hz) oscillation patterns greater than 0.1 Hz [13]. A substantial portion of the fMRI signal obtained during rest and processed through this kind of approach can be attributed to spontaneous BOLD activity compared to that attributable to scanner and physiological artefacts [12].

**Rationales for using different statistical tests for GVM and remaining imaging analyses**

In this study, we used the TFCE-based permutation test as the statistical test of VBM and the standard SPM-based cluster size test for remaining imaging analyses. This is because the standard cluster size test is inappropriate in voxel-based morphometry [14], whereas a permutation-based test is assumed to be correct. However, the abovementioned analysis using BPM cannot be applied to rGMV analysis because the TFCE toolbox cannot be used in BPM, which is why we chose this statistical design for rGMV analysis. SPM8 was used here because of better compatibility of the software of TFCE and home-made script for analyses. As long as TFCE is used, the rationale for estimating the statistical significance is same, and this would not matter to the results.

**References**

[1] S. Nakamura, S. Anderton, M. Kanzaki, and M. Kobayashi. TOEIC(R) test new best triple practice exam. ed. Series Editor, editor^editors.: Japan Times; 2008. of p.

[2] H. Takeuchi, Y. Taki, R. Nouchi et al., "Effects of Multitasking-Training on Gray Matter Structure and Resting State Neural Mechanisms," *Human Brain Mapping*, vol. 35, no. 8, pp. 3646-3660, 2014.

[3] H. W. Mahncke, B. B. Connor, J. Appelman et al., "Memory enhancement in healthy older adults using a brain plasticity-based training program: a randomized, controlled study," *Proceedings of the National Academy of Sciences of the United States of America*, vol. 103, no. 33, pp. 12523-12528, 2006.

[4] H. Takeuchi, Y. Taki, and R. Kawashima, "Effects of working memory training on cognitive functions and neural systems," *Reviews in the Neurosciences*, vol. 21, no. 6, pp. 427-450, 2010.

[5] C. Pantev, M. Hoke, B. Lutkenhoner, and K. Lehnertz, "Tonotopic organization of the auditory cortex: pitch versus frequency representation," *Science*, vol. 246, no. 4929, pp. 486-488, 1989.

[6] P. Adank, and J. T. Devlin, "On-line plasticity in spoken sentence comprehension: Adapting to time-compressed speech," *Neuroimage*, vol. 49, no. 1, pp. 1124-1132, 2010.

[7] H. Takeuchi, Y. Taki, R. Nouchi et al., "Effects of working memory-training on functional connectivity and cerebral blood flow during rest," *Cortex*, vol. 49, no. 8, pp. 2106-2125, 2013.

[8] H. Takeuchi, Y. Taki, R. Nouchi et al., "Working memory training impacts the mean diffusivity in the dopaminergic system.," *Brain Structure and Function*, vol. 220, no. 6, pp. 3101-3111, 2015.

[9] C. Thomas, and C. I. Baker, "Teaching an adult brain new tricks: a critical review of evidence for training-dependent structural plasticity in humans," *Neuroimage*, vol. 73, pp. 225-236, 2012.

[10] M. L. Schölvinck, A. Maier, F. Q. Ye, J. H. Duyn, and D. A. Leopold, "Neural basis of global resting-state fMRI activity," *Proceedings of the National Academy of Sciences*, vol. 107, no. 22, pp. 10238-10243, 2010.

[11] H. Takeuchi, Y. Taki, R. Nouchi et al., "Degree centrality and fractional amplitude of low-frequency oscillations associated with Stroop interference," *Neuroimage*, vol. 119, no. 1, pp. 197-209, 2015.

[12] D. M. Cole, S. M. Smith, and C. F. Beckmann, "Advances and pitfalls in the analysis and interpretation of resting-state FMRI data," *Frontiers in Systems Neuroscience*, vol. 4, pp. 8, 2010.

[13] D. Cordes, V. M. Haughton, K. Arfanakis et al., "Frequencies contributing to functional connectivity in the cerebral cortex in “resting-state” data," *American Journal of Neuroradiology*, vol. 22, no. 7, pp. 1326-1333, 2001.

[14] S. Hayasaka, K. L. Phan, I. Liberzon, K. J. Worsley, and T. E. Nichols, "Nonstationary cluster-size inference with random field and permutation methods," *Neuroimage*, vol. 22, no. 2, pp. 676-687, 2004.
